# Cannabigerol (CBG) signal enhancement in its analysis by gas chromatography coupled with tandem mass spectrometry

**DOI:** 10.1007/s11419-023-00673-x

**Published:** 2023-09-27

**Authors:** Andrzej L. Dawidowicz, Rafal Typek, Michal P. Dybowski, Piotr Holowinski, Michal Rombel

**Affiliations:** Department of Chromatography, Institute of Chemical Sciences, Faculty of Chemistry, Maria Curie-Sklodowska University in Lublin, 20-031 Lublin, Poland

**Keywords:** GC–MS, Signal enhancement, Cannabigerol, Sample derivatization, CBG cyclization

## Abstract

**Purpose:**

According to recent reports, cannabigerol (CBG) concentration level in blood and body fluids may have forensic utility as a highly specific albeit insensitive biomarker of recent cannabis smoking. While the analytical sensitivity of cannabidiol (CBD), Δ^9^-tetrahydrocannabinol (Δ^9^-THC), cannabichromene (CBC) or cannabinol (CBN) estimation by gas chromatography-mass spectrometry (GC–MS) is similar and sufficiently high, it is exceptionally low in the case of CBG (ca. 25 times lower than for the other mentioned cannabinoids). The purpose of this study is to explain the reasons for the extremely low analytical sensitivity of GC–MS in estimating CBG and to present possible ways of its improvement.

**Methods:**

Nuclear magnetic resonance (NMR) data and GC–MS responses to CBG and its various derivatization and transformation products were studied.

**Results:**

The validation data of individual derivatives of CBG and its transformation products were established. CBG silylation/acylation or hydration allows to decrease LOD about 3 times, whereas the formation of pyranic CBG derivative leads to 10-times decrease of LOD. The paper enriches the literature of the subject by providing MS and NMR spectra, not published so far, for derivatives of CBG and its transformation products. The most likely cause of low GC–MS response to CBG is also presented.

**Conclusions:**

The presented results shows that although the signal increase of CBG can be obtained through its derivatization by silylation and/or acylation, the greatest increase is observed in the case of its cyclization to the pyranic CBG form during the sample preparation process. The CBG cyclization procedure is very simple and workable in estimating this cannabinoid in blood/plasma samples.

**Graphical abstract:**

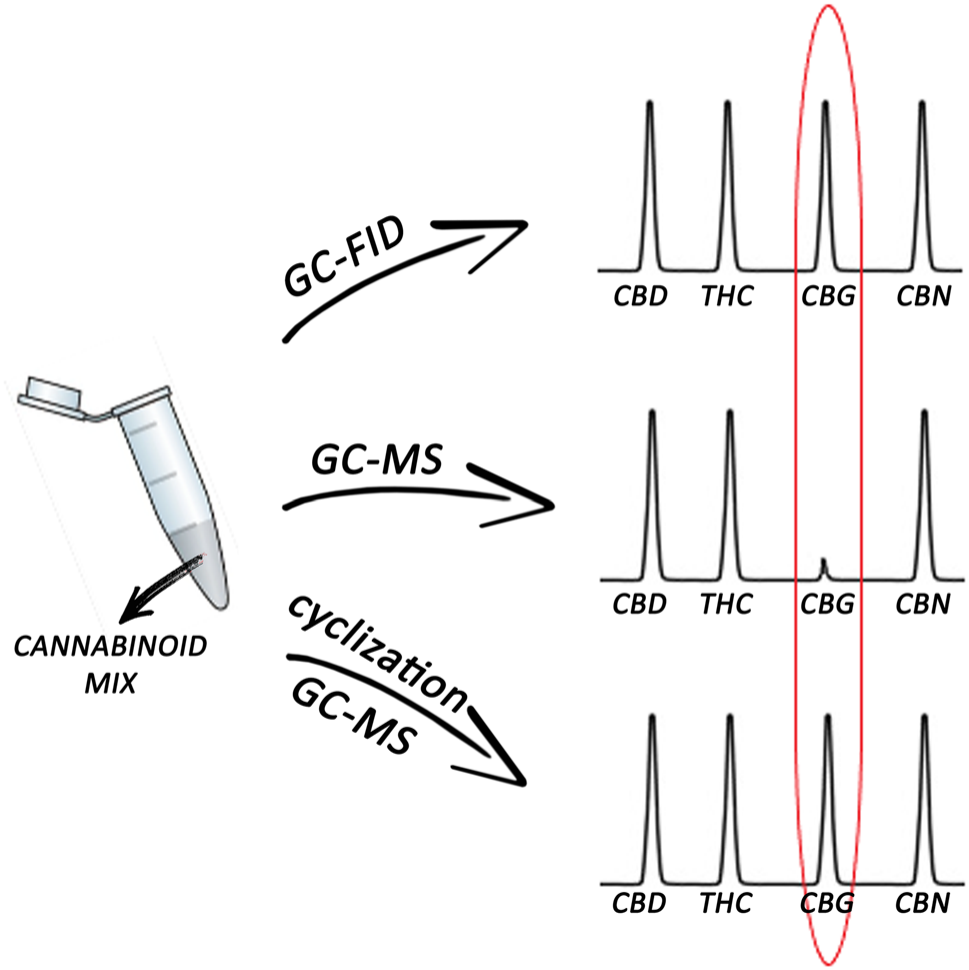

**Supplementary Information:**

The online version contains supplementary material available at 10.1007/s11419-023-00673-x.

## Introduction

Cannabinoids constitute a group of at least 150 diverse organic compounds exhibiting bioactive properties due to their ability to interact with CB1 and CB2 receptors of the endocannabinoid system that is very important one for various health systems [1–3]. Although psychoactive Δ^9^-tetrahydrocannabinol (Δ^9^-THC) is still the most famous cannabinoid in this group [4–6], cannabidiol (CBD), devoid of psychotropic effect, has become nowadays just as popular due to its medicinal properties [7, 8]. In addition to its potential activity in the treatment of epileptic syndromes [9–11], preclinical studies demonstrate wider bio-activity of CBD, suggesting its possible usage in the treatment of many other disease entities [12, 13]. Lately, more and more attention has also been paid to the bioactive properties of another cannabinoid, cannabigerol (CBG). The literature on its bioactive properties is not as extensive as on CBD, but it indicates, *inter alia*, its cytotoxic effect against epithelioid carcinoma and breast cancer, and its stimulating effect on a number of receptors important for pain, inflammations, and heat sensitization [14]. Recently, CBG has also become an interesting object for forensic analysis researchers. The reports [15–18] show that it may be treated as a highly specific, though insensitive, bio-marker of recent cannabis smoking. Because Δ^9^-THC can persist in the blood after the psychoactive effects of intoxication have subsided, especially in regular users, short-lived minor cannabinoids such as CBG have merited investigation as additional indicators of recent cannabis inhalation. Another reason for the interest of forensic analysts in the determination of CBG in physiological fluids is related to its alpha-2-adrenergic action promoting hypotension and bradycardia [19], what can cause fatal descent.

Growing interest in the mentioned cannabinoids and their bioactive properties, as well as a marked increase in the use of dietary supplements containing these cannabinoids in self-healing therapies [20], require the development of reliable and sensitive analytical procedures for their quantitative determination in different types of matrices. While both gas chromatography (GC) or high-performance liquid chromatography (HPLC) can be used for this purpose, GC is a more pragmatic option as the choice a simpler, cheaper and a more sensitive technique. In this technique, gas chromatography-tandem mass spectrometry (GC–MS/MS) is becoming a routine analytical instrument, advisable especially when analyzing compounds in very complex matrices (e.g. in plasma/plant material) [21–23].

A very important feature of any analytical procedure is its sensitivity towards the examined compound. In forensic and clinical analysis, high sensitivity analytical methods are usually preferred. The more sensitive the analytical method, the lower concentration level of given xenobiotic can be determined in examined sample. As results from the previous reports [24, 25] and our experiences, the sensitivity of analytical estimation of CBD and Δ^9^-THC (or such cannabinoids like cannabichromene (CBC) and cannabinol (CBN)) using GC–MS is similar and sufficiently high, but it is exceptionally low for CBG. The aim of this study is to explain the reasons for the extremely low analytical sensitivity of GC–MS in estimating CBG and to present possible ways of its improvement.

## Materials and methods

### Materials

Acetonitrile (ACN) (LC/MS grade), the standards (certified reference materials) of CBC, CBN, CBG, CBD and Δ^9^-THC (1.0 mg/mL in methanol—Cerilliant), trifluoroacetic anhydride (TFAA), hexamethyldisilazane (HMDS) and trimethylchlorosilane (TMCS), palladium on carbon (Pd/C) and p-toluenesulfonic acid (PTSA) were acquired from Merck (Merck KGaA, Darmstadt, Germany). Dichloromethane (DCM) and sodium dicarbonate were purchased from the Polish Chemical Plant POCh (Gliwice, Poland). CDCl_3_ was purchased from Armar AG (Döttingen, Switzerland). CBG crystal (> 99%) was a gift from CannLAB (Kraków, Poland). Hydrogen and nitrogen were obtained from Air Liquide Polska (Kraków, Poland). Deionized water was purified by the Milli-Q system (Merck Millipore, Merck KGaA, Darmstadt, Germany).

Human plasma samples were obtained by the centrifugation of human blood samples. The samples were collected by a registered nurse from volunteers, after obtaining their informed consent, using a single closed system containing an S-Monovette coagulation activator (Citrate 3.2%), following the manufacturer’s instructions (Sarstedt AG, Nümbrecht, Germany), and thoroughly mixed in order to maintain their homogeneity. The plasma samples were CBG free.

### CBG silylation procedure (*formation of CBG-2TMS*)

The CBG silylation was carried out according to the optimal procedure described in the reports [25, 26]. The mixture consisting of 500 μL of CBG solution in ACN (1 mg/mL) and 500 μL of silylation mixture (HMDS/TMCS/ACN 1:1:1 v/v/v) was heated at 35 °C for 60 min. The mixture was subsequently centrifuged at 12,000 rpm for 5 min and the obtained supernatant was divided into two parts. One part was diluted 500 times using ACN and subjected to GC/MS analysis. The other one was evaporated under nitrogen stream, dissolved in CDCl_3_ (500 μL) and subjected to nuclear magnetic resonance (NMR) analysis.

### CBG acylation procedure by TFAA (*formation of CBG-2TFA*)

The CBG acylation was carried out according to the optimal procedure described in the reports [27, 28]. The mixture consisting of 500 μL of CBG solution in ACN (1 mg/mL) and TFAA (130 μL) was heated at 65 °C for 60 min. After that, the high volatile components of the reaction mixture were evaporated under nitrogen stream and the obtained dry residue was dissolved in an appropriate solvent before GC/MS and NMR analysis. In GC/MS analysis, the dry residue was dissolved in ACN (1 mL) and then diluted 1000 times. In NMR analysis, the dry residue was dissolved in CDCl_3_ (500 μL).

### CBG hydrogenation (formation of CBG-4H)

The suspensions consisting of Pd/C (1 mg) and 1 mL of CBG solution in DCM (5 mg/mL) were mixed for 3 h in hydrogen atmosphere at ambient temperature and then centrifuged at 12,000 rpm for 2 min. The obtained supernatant, before its GC/MS analysis, was diluted 5000 times using DCM, whereas before NMR analysis, the supernatant was evaporated to dryness and the obtained dry residue was dissolved in CDCl_3_ (500 μL). The above procedure is the result of optimizing the time and temperature of the process.

### Formation of pyranic CBG structure (*Pyr-CBG*) in chemical process (chemical CBG cyclization)

The mixture consisting of 500 μL of CBG solution in ACN (5 mg/mL) and 500 μL of PTSA solution in ACN (1 mg/mL) was heated at 75 °C for 2 h. In the case of the reaction product estimation by GC–MS, the obtained mixture was diluted 2500 times using ACN and subjected to GC–MS analysis. The above procedure was optimized studying the influence of time and temperature on the course of CBG cyclization in its ACN solution.

The estimation of the reaction product by NMR requires the removal of PTSA from the mixture. To do this, the obtained mixture was evaporated to dryness under nitrogen stream. Next, the dry residue was dissolved in DCM (500 μL) and extracted three times by the saturated water solution of NaHCO_3_ (500 μL), removing water phase each time. Finally, the cleaned organic phase was evaporated under nitrogen stream and the obtained dry residue was dissolved in CDCl_3_ (500 μL).

### Silylation of pyranic CBG structure (formation of Pyr-CBG-TMS)

The mixture consisting of 500 μL of CBG solution in ACN (5 mg/mL) and 500 μL of PTSA solution in ACN (1 mg/mL) was heated at 75 °C for 2 h and then evaporated to dryness under nitrogen stream. Next, the dry residue was dissolved in DCM (500 μL) and extracted 3 times by saturated water solution of NaHCO_3_ (3 × 500 μL), removing water phase each time. Finally, the cleaned organic phase was evaporated under nitrogen stream and the obtained dry residue was silylated by heating at 35 °C for 60 min with 1 mL of silylation mixture (HMDS/TMCS/ACN 1:1:1 v/v/v). The mixture was subsequently centrifuged at 12,000 rpm for 5 min and the obtained supernatant, after its prior 2500 fold dilution by ACN, was subjected to GC–MS analysis.

### Formation of pyranic CBG structure in physical processes (physical CBG cyclization)

To find out if an energetic physical action on CBG can transform it into its pyrenic form, separate CBG samples were exposed to high temperature, UV and electron stream radiation.

*CBG thermal treatment* Thermal treatment of the CBG sample was performed in a closed glass vessel containing argon atmosphere at 270 °C for 2 h using a classical laboratory oven.

*CBG exposion to UV radiation* The CBG sample, placed in a thin quartz tube, was exposed to UV radiation for 5 min using a high energetic UV lamp emitting 2.0 kW energy.

*CBG exposion to electron stream radiation* The CBG sample was exposed to electron stream for 10 h using the electron source of energy 7 eV. UHV Flood Gun (PreVac, Rogów, Poland) was used for this purpose.

After the physical treatment, each sample was dissolved in DCM and subjected to GC–MS analysis.

### Preparation of plasma samples for GC–MS analysis of CBG, CBN, CBD, Δ9-THC and CBC mixture using QuEChERS procedure

#### The version without CBG cyclization

MgSO_4_ (200 mg) and NaCl (50 mg) were added to a plasma sample (700 μL) spiked properly with the mixture of CBG/CBN/CBC/CBD/Δ^9^-THC. The concentration of individual cannabinoids (CBG, CBN, CBD, Δ^9^-THC and CBC) was 20 ng/mL, similar to the concentration of that determined in the real blood samples from cannabis/hemp consumers [29, 30]. After vortexing for 1 min, ACN (700 μL) was introduced and the whole suspension was vortexed again and then centrifuged at 12,000 rpm for 3 min. The isolated aliquot was subjected to GC–MS analysis.

#### The version with CBG cyclization

MgSO_4_ (200 mg) and NaCl (50 mg) were added to a plasma sample (700 μL) spiked properly with the mixture of CBG/CBN/CBC/CBD/Δ^9^-THC—the concentrations of individual cannabinoids were the same as in the version without CBG cyclization. After vortexing for 1 min, 700 μL of PTSA solution in ACN (0.5 mg/mL) was introduced and the whole suspension was vortexed again and then centrifuged at 12,000 rpm for 3 min. The isolated aliquot (600 μL) was heated at 75 °C for 2 h. After cooling, the obtained solution was subjected to GC–MS analysis.

### GC-FID measurements

ACN solution of CBG, CBD, THC, CBC and CBN mixture was analysed using gas chromatograph with the flame ionization detector GC-FID model GC-2010 (Shimadzu, Kyoto, Japan). 1 μL sample was injected by an AOC–20i type autosampler into a ZB5-MS fused-silica capillary column (30 m × 0.25 mm i.d., 0.25 μm film thickness) (Phenomenex, USA). Separation conditions were the following: carrier gas—hydrogen (grade 5.0); flow rate—1.0 ml/min; split injection mode; injector temperature—310 °C. The temperature program involved: initial temperature 150 °C held for 5 min; temperature increase to 260 °C (at a rate of 9 °C/min) and maintained for 4 min; further temperature increase to 300 °C (at a rate of 6 °C/min).

### GC–MS/MS measurements

Qualitative analyses of the examined cannabinoids and CBG transformation products were conducted using a gas chromatograph hyphenated with a triple quadrupole tandem mass spectrometer detector (GCMS-TQ8040; Shimadzu, Kyoto, Japan). GC–MS conditions were as follows: capillary column—Zebron ZB5-MSi (30 m × 0.25 mm i.d., 0.25 μm film thickness, Phenomenex, Torrance, CA, USA); carrier gas helium (grade 5.0); flow rate 1.0 ml/min; splitless/split injection mode (sampling time: 1.00 min); injector temperature 310 °C; injection volume 1 μL; temperature program—initial temperature 60 °C held for 3 min subsequently increased to 310 °C at the rate of 12 °C/min and held for 15 min. Mass spectrometer parameters: normalized electron energy of 70 eV; ion source temperature 225 °C. The TIC mode with range 40–750 *m/z *was used in analysing solutions of cannabinoids and CBG transformation products. In order to analyse QuEChERS extracts from the supernatants centrifuged from human plasma samples spiked with CBG, CBN, CBD, Δ9-THC and CBC, multiple reaction monitoring (MRM) mode was used. GC–MS/MS analysis was performed using characteristic MRM transitions at optimal collision energies (CE) for the examined compounds. Three MRM transitions (*m/z* =  > *m/z*) of the highest intensity were selected for further experiments:

314 > 231 (CE = 24 eV), 314 > 193 (CE = 21 eV) and **246 > 231** (CE = 21 eV) for CBD,

**316 > 231** (CE = 15 eV), 316 > 193 (CE = 15 eV) and 316 > 123 (CE = 6 eV) for CBG,

**314 > 299** (CE = 12 eV), 314 > 271 (CE = 15 eV) and 314 > 231 (CE = 18 eV) for Δ^9^-THC,

**314 > 299** (CE = 12 eV), 314 > 271 (CE = 15 eV) and 314 > 231 (CE = 18 eV) for Δ^8^-THC,

**316 > 231** (CE = 15 eV), 316 > 193 (CE = 15 eV) and 316 > 123 (CE = 6 eV) for Pyr-CBG,

314 > 299 (CE = 15 eV), **314 > 231** (CE = 15 eV) and 299 > 231 (CE = 6 eV) for CBC,

310 > 295 (CE = 21 eV), 310 > 238 (CE = 30 eV) and **295 > 238** (CE = 27 eV) for CBN.

MRM transitions selected for quantification of individual cannabinoids were bolded and underlined.

### NMR measurements

NMR experiments were performed using the Ascend 600 MHz instrument (Bruker, Bremen, Germany) equipped with 5 mm BBO probe at 298 K. Before 1H and 13C spectra acquisition, examined samples were dissolved in CDCl_3_. ^1^H–^1^H Double Quantum Filtered COSY and ^1^H–^13^C Multiplicity Edited HSQC techniques were employed to facilitate assignment of proton and carbon resonances. Acquisition and processing of considered spectra were performed using TopSpin 3.5 and IconNMR software (Bruker, Bremen, Germany). Tetramethylsilane was used as an internal standard for calibration of ^1^H and ^13^C chemical shifts. NMR data are gathered in Table 1S and 2S (see supplementary materials).

### Method validation

To validate the analytical methods, certified standards of individual CBG derivatives and Pyr-CBG should be purchased. Such substances are not commercially available. For this reason, purified CBG derivatives and Pyr-CBG obtained for NMR analysis were used as validation standards. Their purity was 95.3, 96.4, 99.2 and 98.7% for CBG-2TMS, CBG-2TFA, CBG-4H and Pyr-CBG, respectively. These values were estimated by GC–MS using peak normalization method. The compounds used for methods validation should be treated as Type II standards.

The methods were validated in terms of linearity, the limit of detection (LOD), the limit of quantification (LOQ) and the intraday and interday precision and accuracy measurements. To evaluate the method linearity, five replicated analytical procedures were performed for each examined concentration level. The LOD and LOQ were considered to be signal-to-noise ratios equal to 3 and 10, respectively. The intra- and interday precisions and accuracies were evaluated by statistical analysis of the quantitative results (obtained on the same day and on three different days) for five independent samples containing test compounds (20 ng/mL). The linearity of the assay was calculated by the least squares method and expressed as the coefficient of determination (*R*^2^). Calibration plots were prepared using ACN spiked with the target analytes at concentration levels of 10, 25, 50, 75 and 100 ng/mL for CBG, 5, 10, 25, 50 and 75 ng/mL for CBG-2TMS, CBG-2TFA and CBG-4H, and 1, 2.5, 5, 10, 25 ng/mL for Pyr-CBG. The solutions were prepared in triplicate.

## Results

**Fig. 1:**
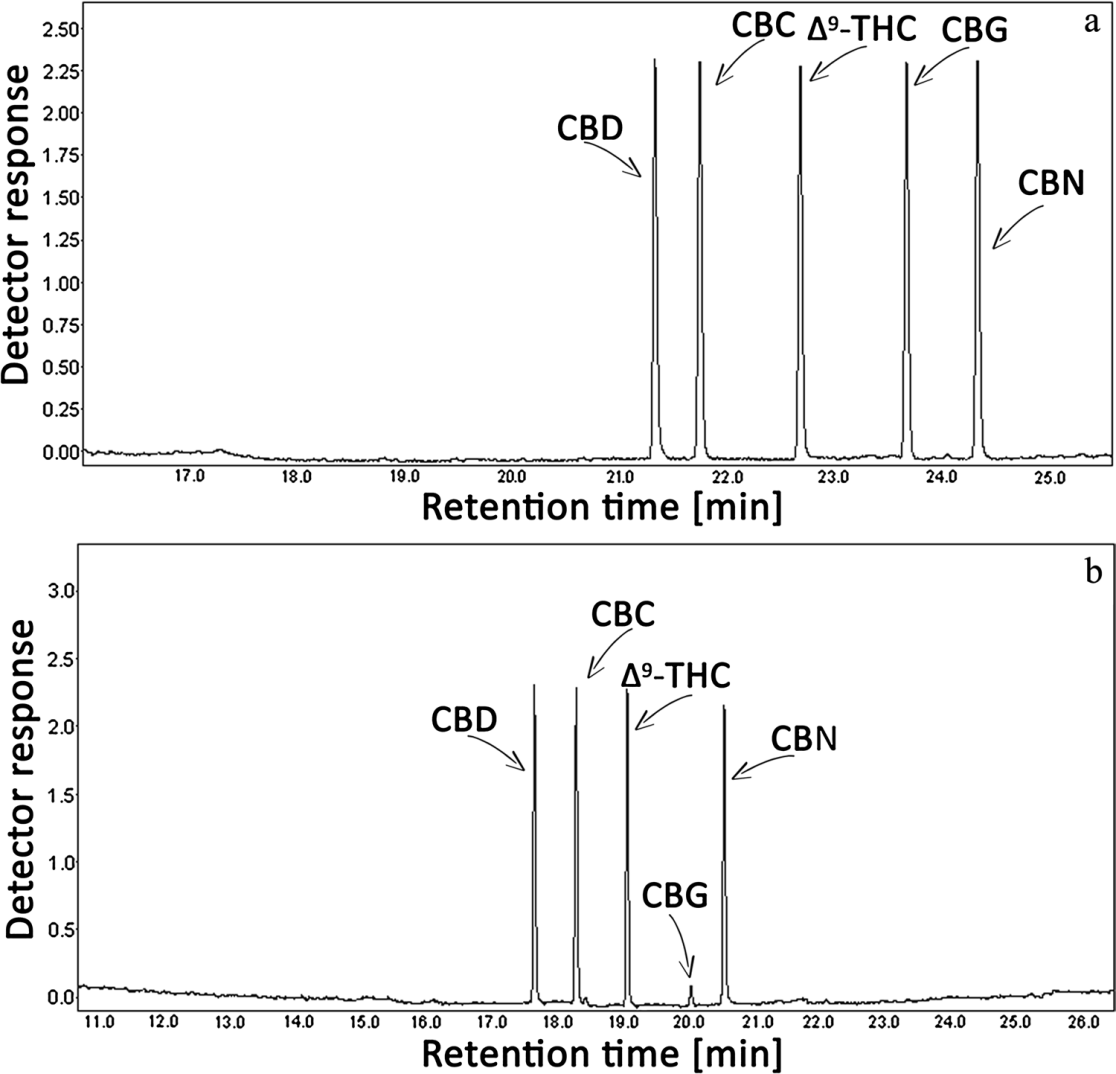
Exemplary chromatogram obtained by GC-FID (**a**) and total ion current chromatogram (TICC) by GC-MS (**b**) of CBG, CBD, CBC, Δ9-THC and CBN mixture dissolved in ACN. The molar concentration of each mixture component was the same and amounted 0.5 mM.

Figure 1 shows exemplary chromatograms of CBG, CBD, CBC, Δ^9^-THC and CBN mixture obtained using GC with FID and MS detector (a and b, respectively). The molar concentrations of the components in the mixture were the same (0.5 mM). As can be seen, the GC–MS response for CBD, CBC, Δ^9^-THC or CBN is similar and high, it is exceptionally low in the case of CBG (ca. 25 times lower than for the other mentioned cannabinoids).

**Fig. 2:**
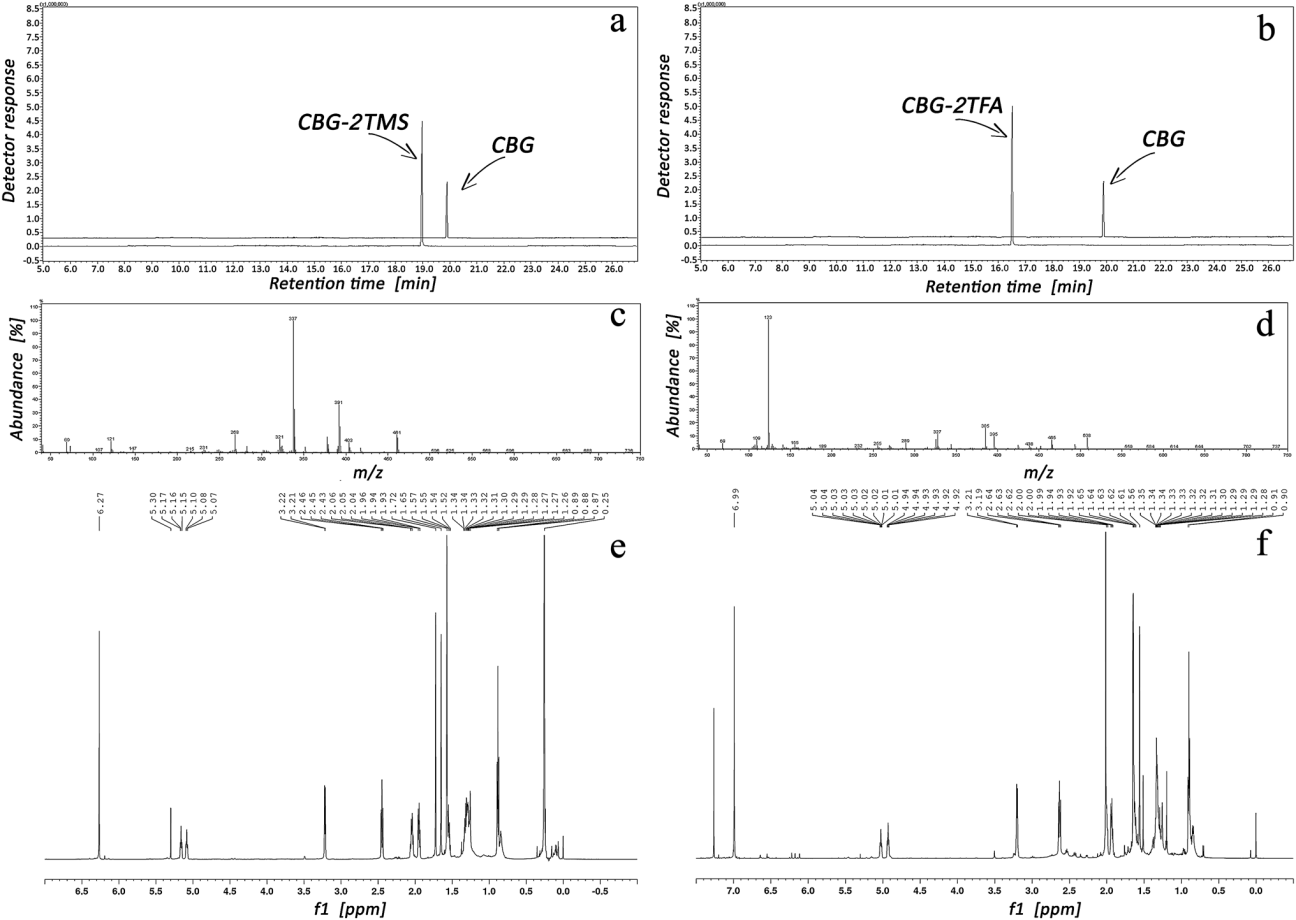
TICCs of CBG and CBG-2TMS (**a**), CBG and CBG-2TFA (**b**) corresponding before and after derivatization, respectively. MS spectra of CBG-2TMS (**c**) and CBG-2TFA (**d**). NMR spectra of CBG-2TMS (**e**) and CBG-2TFA (**f**).

The most frequent way of increasing the chromatographic analysis sensitivity to the examined compounds is their preliminary derivatization, usually performed by silylation or acylation. Figure 2 shows the size of the GC–MS response to CBG itself and to its silyl and acyl derivative obtained after CBG silylation with TMCS/HMDS (a) and acylation with TFAA (b). MS and NMR spectra of the obtained TMS derivative of CBG are provided in c and e, respectively, and of the obtained TFA derivatives of CBG in d and f, respectively.

**Fig. 3:**
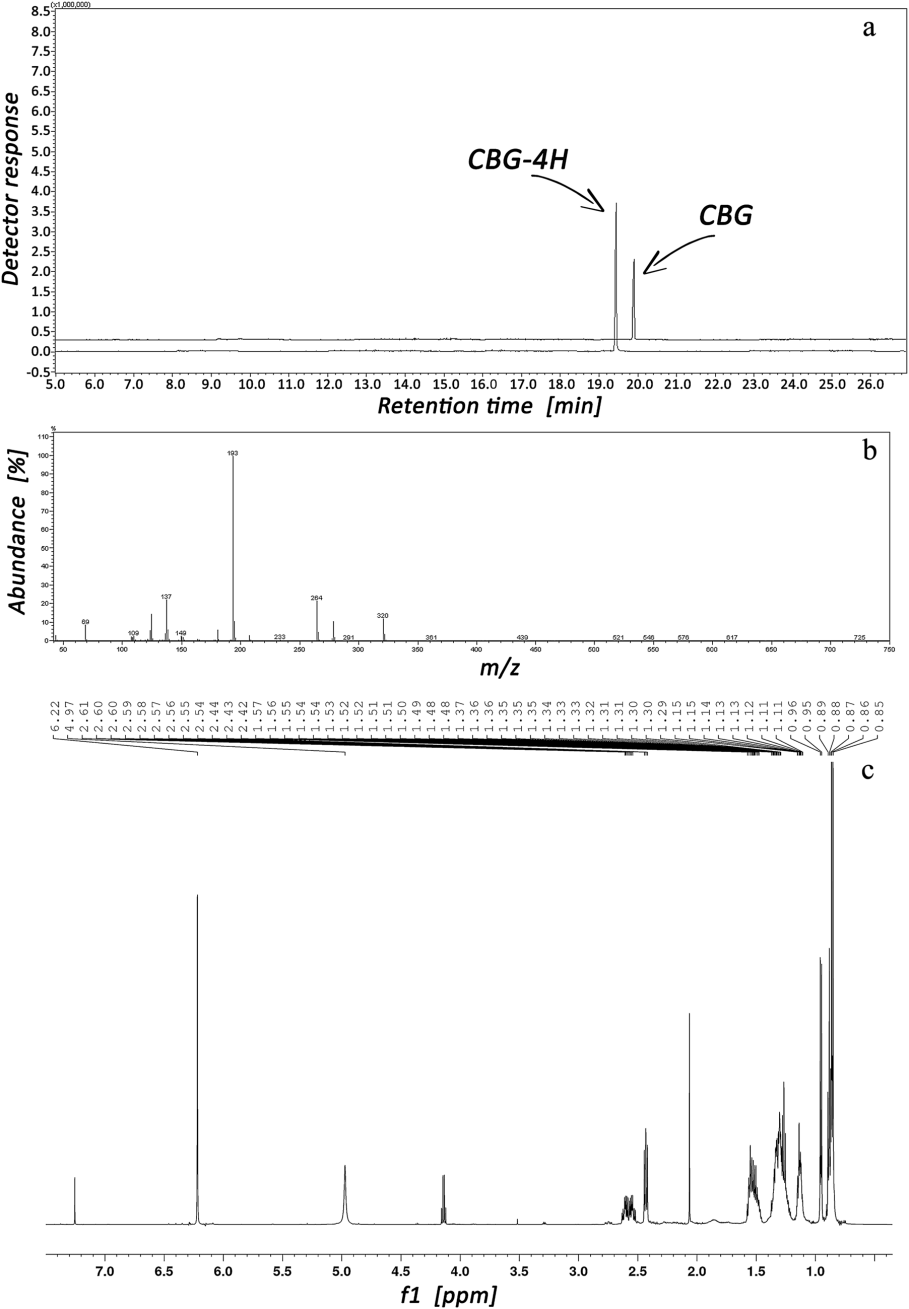
TICCs of CBG and CBG-4H corresponding before and after hydrogenation (**a**). MS spectrum (**b**) and NMR spectrum (**c**) of CBG-4H, respectively.

The size change of the GC–MS signal resulting from the saturation of alkyl-diene chain in the CBG molecule by hydrogen is shown in Fig. 3a, whereas Fig. 3b and c present, respectively, MS and NMR spectra of the obtained saturated derivative of CBG.

**Fig. 4:**
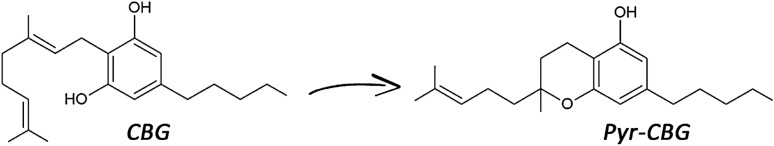
Cyclization reaction of CBG to its pyranic form (Pyr-CBG).

**Fig. 5:**
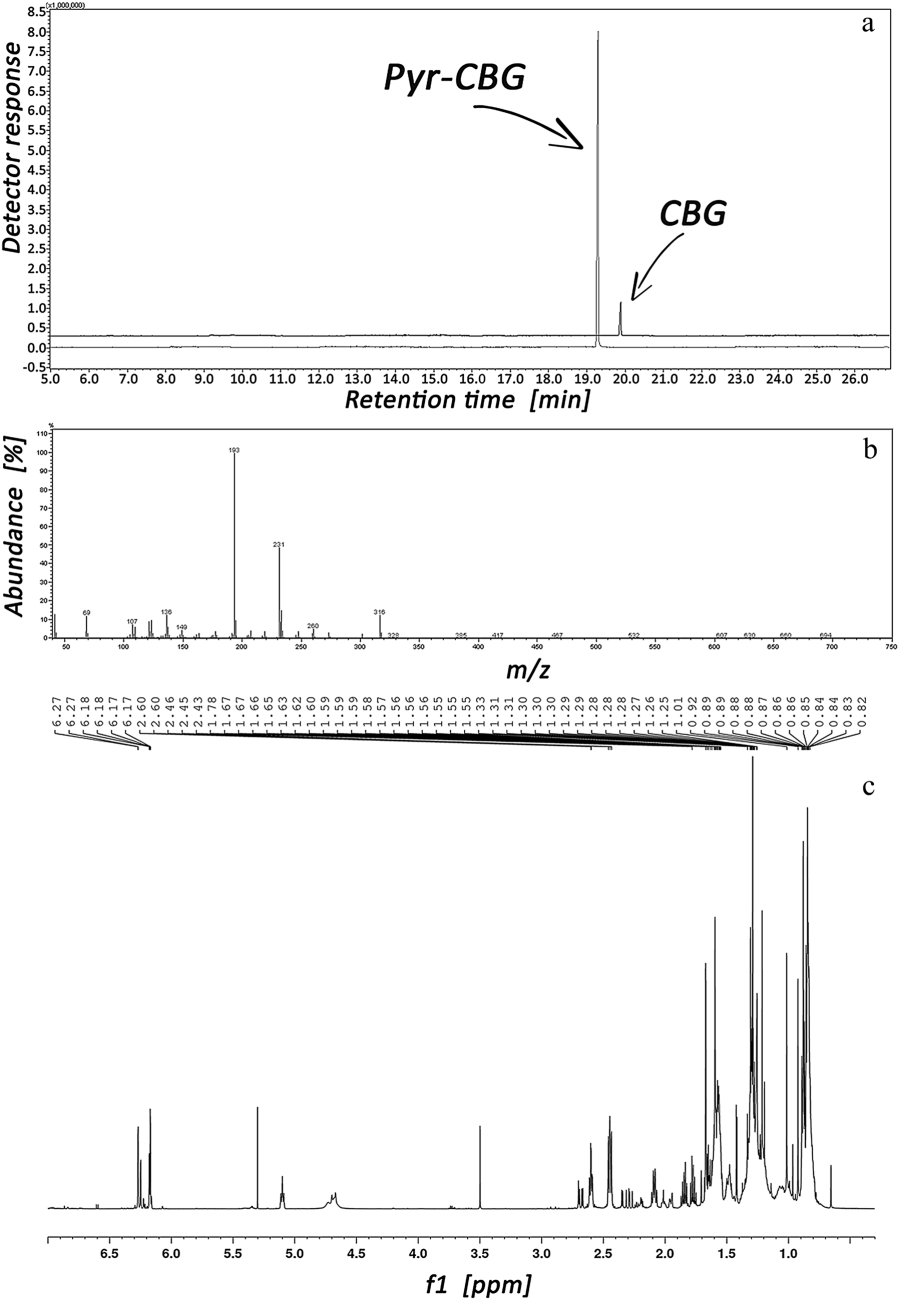
TICCs of CBG and Pyr-CBG corresponding before and after cyclization (**a**). MS spectrum (**b**) and NMR spectrum (**c**) of Pyr-CBG, respectively.

The presence of alkyl-diene chain and phenolic groups in CBG allows the formation of its pyranic derivative (Pyr-CBG). The diagram of such a transformation illustrates Fig. 4. The change in the GC–MS signal size resulting from CBG transformation into Pyr-CBG is presented in Fig. 5a, whereas MS and NMR spectra of this compound are shown in Fig. 5b and c, respectively.

Figure 1S (see supplementary materials) shows the chromatograms of DCM solutions of the CBG samples exposed to high temperature, UV and electron stream radiation. These chromatograms indicates that CBG can be cyclized to its pyranic form by energetic physical action.

According to the results presented in Figs. 2a and 5a, the silylation of CBG increases the sensitivity of its GC–MS estimation about 3 times, whereas the cyclization of CBG about 9 times. Figure 2S (see Supplementary Materials) shows GC–MS response to pyranic derivative of CBG before and after its silylation by TMCS/HMDS mixture.

The validation data for CBG, CBG derivatives (CBG-2TMS, CBG-2TFA, CBG-4H) and Pyr-CBG are gathered in Table 1.

**Table 1:** Linearities, intra- and interday precisions and accuracies, limits of detection (LOD), limits of quantification (LOQ) of CBG, CBG-2TMS, CBG-2TFA, CBG-4H and Pyr-CBG, respectively

Tested parameter	Compound				
	CBG	CBG-2TMS	CBG-2TFA	CBG-4H	Pyr-CBG
Linearity (*R*^2^)	0.9991	0.9969	0.9975	0.9988	0.9987
Intraday precision (% RSD)	3.17	4.56	4.61	3.74	3.88
Interday precision (% RSD)	3.32	4.82	4.79	3.95	4.21
Intraday accuracy (%)	98.7	97.9	97.1	98.2	97.9
Interday accuracy (%)	97.6	96.2	96.6	97.1	96.9
LOD (ng/mL)	2.78	0.93	0.91	0.83	0.26
LOQ (ng/mL)	9.26	3.09	3.03	2.76	0.86

*R*^2^ coefficient of determination, *RSD* relative standard deviation, *LOD* limit of detection, *LOQ* limit of quantification

**Fig. 6:**
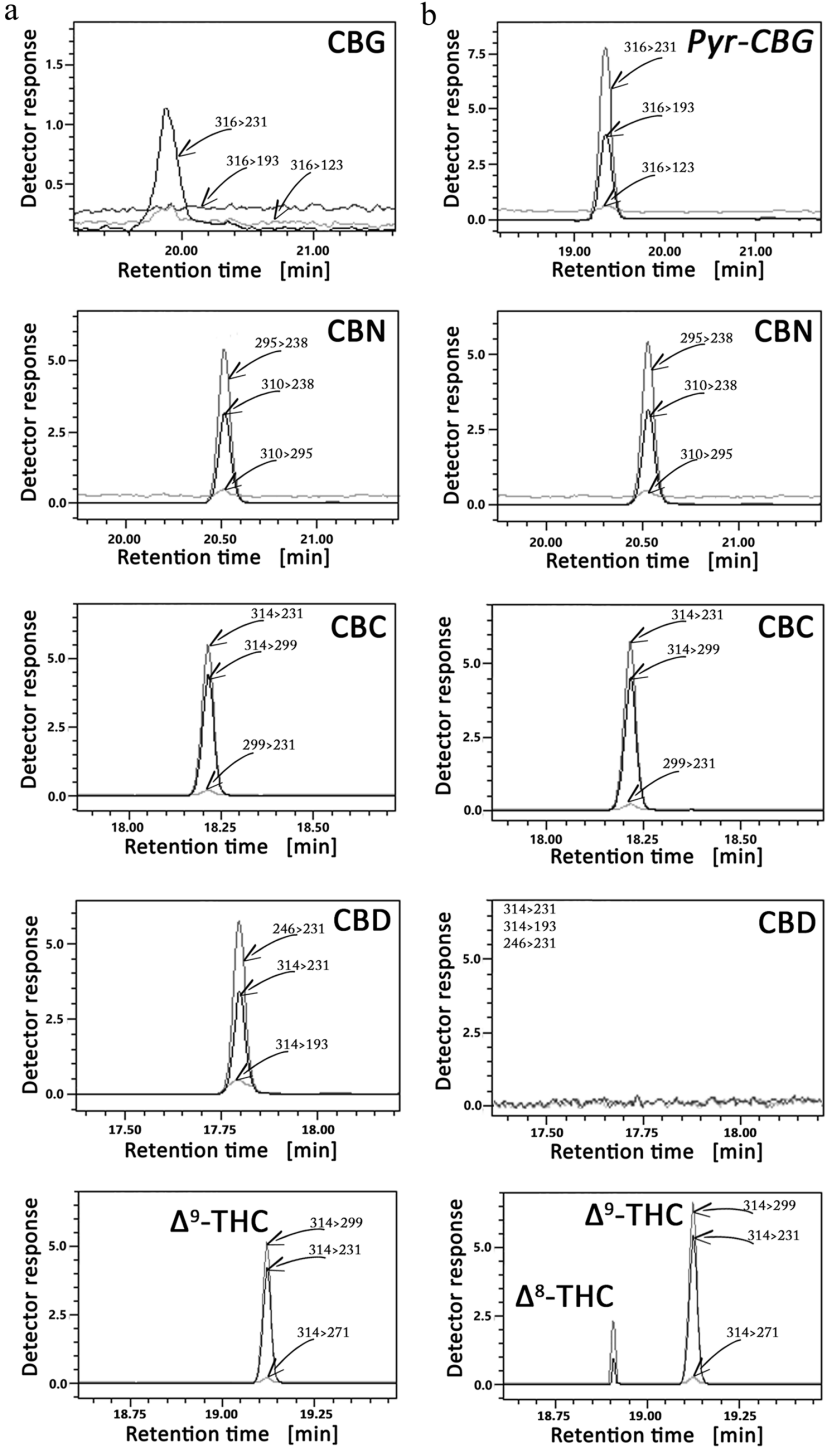
MRM chromatograms of blood sample containing CBG, CBN, CBD, Δ9-THC and CBC mixture before (**a**) and after cyclization (**b**). Arrows indicate signals with corresponding MRM transitions.

The practical utility of the signal enhancement of CBG by its cyclization is shown in Fig. 6 illustrating the results of GC–MS analyses of CBG, CBN, CBD, Δ^9^-THC and CBC mixture in plasma samples. Classical QuEChERS and QuEChERS with the CBG cyclization process were used as blood sample preparation methods in the analytical experiments.

## Discussion

As results from Fig. 1 showing exemplary chromatograms of CBG, CBD, CBC, Δ^9^-THC and CBN mixture obtained by GC with FID and MS detector, FID responses to individual cannabinoids are similar. This is not a surprising effect in the analysed compounds and results from the same number of carbon atoms in each of them and from their structural similarity. A slightly different chromatographic image of the tested mixture is observed in GC–MS. While the MS responses to CBD, CBC, Δ^9^-THC and CBN are similar, the MS signal for CBG is extremely low (ca. 25 times lower than for the other mentioned cannabinoids), which confirms experimentally our earlier observations and sparse literature reports [24, 25] about poor response of the MS detector to CBG.

The most frequent way of increasing the chromatographic analysis sensitivity to the examined compounds is their preliminary derivatization, usually performed by silylation or acylation. As can be seen from the spectroscopic data presented in Fig. 2c–f, the use of excessive amounts of silylating or acylating reagents in the CBG derivatization process, which is common practice in the case of the same processes with other compounds, leads to the derivatization of both phenolic groups of this cannabinoid. As seen in Fig. 2a and b, the silyl and acyl derivative of CBG evokes a GC–MS signal about 3 times higher than unmodified CBG. Thus, these ways of CBG derivatization allow to lower LOD in the analytical estimation of CBG (see Table 1), but it still remains much greater than the LOD for non-derivatized CBD, CBC, Δ^9^-THC or CBN. In the case of the equipment used in these experiments, LOD for the last mentioned cannabinoids does not exceed 0.1 ng/mL. It is worth noting here that the figure also enriches the literature of the subject by providing MS and NMR spectra of the obtained CBG-2TMS derivatives (c and e, respectively) and CBG-2TFA derivatives (d and f, respectively).

A characteristic feature of the CBG molecule, in contrast to CBD, CBC, Δ^9^-THC and CBN, is the presence of an alkyl-diene chain. The saturation of multiple bonds in the analyte with hydrogen is difficult to classify as a classical derivatization procedure, but it was intended to raise the CBG signal in GC–MS analysis. According to NMR data presented in Fig. 3c and Table 1S (see Supplementary Materials), 3-h hydrogenation process of CBG provides total saturation of this cannabinoid—the obtained saturated CBG derivative (CBG-4H) does not contain double bonds in alkyl chain. As it can be seen from Fig. 3a, GC–MS signal increment resulting from the saturation of both double bonds in alkyl-diene chain of CBG is similar to that observed after CBG silylation and/or acylation, i.e. the saturated derivate of CBG (i.e. CBG-4H) evokes a GC–MS signal about 3 times higher than unmodified CBG. However, this effect is much less practical as it was achieved after a 3-h saturation process, much longer than the 1-h silylation/acylation classical derivatization.

Due to the presence of alkyl-diene chain and phenolic groups, CBG can be transformed into its pyranic form (see Fig. 4). The molecular structure of this transformation product is more similar to the molecular structure of CBD, CBC, Δ^9^-THC and CBN than to CBG. Following the results presented in Fig. 5a, CBG cyclization evokes ca. ninefold increase of the GC–MS signal, significantly higher than classical CBG derivatization (silylation/acylation) or CBG saturation. The pyranic derivative of CBG presented in this study is a new compound obtained by cyclization of CBG, and this is why it can be call either cyclo-CBG or pyro-CBG (because it has a pyran ring). The structure of the obtained Pyr-CBG, presented in Fig. 4, is confirmed by NMR data (Fig. 5c and Table 2S in Supplementary Materials). It is worth noting here that another cyclic derivative of CBG has been described in the literature [31], resulting from the metabolism of CBG by human Cytochrome P450s, which was briefly called cyclo-CBG. It should have been named hydroxycyclo-CBG or furan-CBG because it has a furan ring. To avoid confusion, the cyclic derivative of CBG identified and synthetized by us will be referred to as Pyr-CBG in the further part of this paper.

A more detailed analysis of the results shows that the MS spectrum of Pyr-CBG (see Fig. 5b) is almost identical to the MS spectrum of CBG itself (see MS library). Various intensity of the CBG molecular ion is the only difference in these spectra, and it is visibly higher in the spectrum of Pyr-CBG. These findings suggest that when CBG is analysed, all its molecules entering into the ionization chamber are transformed to its pyranic form. Only a small part of the molecules of this compound undergo ionization and fragmentation processes evoking low GC–MS response. It can be assumed that when CBG is analyzed, most of the energy from the EI source is consumed in its cyclization process. Hence, the GC–MS signal from its pyranic structure is greater than that of CBG itself (see Fig. 5a), as all the energy of the EI source is used to ionize Pyr-CBG molecules.

To confirm that CBG can be cyclized to its pyranic form by energetic physical action, separate CBG samples were exposed to high temperature, UV and electron stream radiation. As can be seen from Fig. 1S (see Supplementary Materials), UV and electron stream radiation cause CBG transformation to its pyranic form, which does not happen in the thermally treated CBG sample. It is worth noting that only a small part of the thermally treated CBG sample dissolves in DCM, and that the GC–MS signal of its solution is lower than that of the solution of the unheated CBG sample. These observations are probably connected with CBG polymerization and transformation to CBC [32] during its heating process – see CBC peak in Fig. 1Sa (see supplementary materials). Taking into account the results of two other experiments, CBG exposures to UV and electron stream, CBG transformation to the pyranic form in the GC–MS ionization chamber is very likely.

According to the results presented in Figs. 2a and 5a, the silylation of CBG increases the sensitivity of its GC–MS estimation about 3 times, whereas the cyclization of CBG about 9 times. NMR data for Pyr-CBG (Fig. 5c and Table 2S in supplementary materials) prove that it has one free phenolic group (see Fig. 4), which enables a two-step CBG transformation: cyclization (step one) and silylation/acylation (step two). The related question arises whether, for example, the silylation of Pyr-CBG will make CBG analysis sensitivity higher than cyclization of CBG itself? As follows from Fig. 2S (see Supplementary Materials), showing GC–MS responses to Pyr-CBG before and after its silylation, the silylation process in this particular case does not lead to the increase of the GC–MS signal.

The presence of 3,7-dimethylocta-2,6-dien chain in the CBG molecule allows also for the Diels–Alder reaction to be carried out and to form six other transformation products of this cannabinoid. The possible products of CBG transformation which can be obtained in the Diels–Alder reaction are shown in Fig. 3S (see Supplementary Materials). Diels–Alder reaction was not tested in the present study, because the conditions required for its performance (high temperature and expensive catalyst) make it impractical in the routine CBG analyses in blood/plasma samples.

As results from the performed experiments, the greatest sensitivity of CBG estimation by GC–MS is observed in the procedure involving cyclization of the cannabinoid to its pyranic form (see Table 1). Figure 6 showing the results of GC–MS analyses of plasma samples containing CBG, CBN, CBD, Δ^9^-THC and CBC mixture proves the practical utility of the CBG cyclization. The comparison of the chromatograms of supernatants from plasma samples obtained by classical QuEChERS and by QuEChERS with the CBG cyclization process, which were used as sample preparation methods, indicates that the novel procedure of CBG transformation is applicable in the analytical procedures of real samples. The presented figure shows that the cyclization process causes:

- a multiple increase of the CBG signal;
- remaining of CBC and CBN signals at the same level;
- the decrease of CBD signal. It is obvious as the cyclization of CBG to its pyranic form requires an acidic environment, in which CBD transform to Δ^9^-/Δ^8^-THC [23]
- the increase of Δ^9^-THC signal, which results from CBD transformation in acidic environment (see above comment)
- the appearance of Δ^8^-THC signal, which results from CBD transformation to Δ^9^-/Δ^8^-THC and Δ^9^-THC isomerization to Δ^8^-THC in acidic environment (see above comments)

Although the presented chromatograms indicate that the cyclization process causes an additional CBD and THC transformations, nevertheless, the method has great potential in increasing the CBG signal making the quantification of this bio-marker in real samples easier.

## Conclusions

It is suggested that due to the relatively rapid metabolism of CBG, the estimation of its concentration in blood or oral fluid can be used to determine cannabis use. A low CBG signal in its GC–MS analysis seriously limits accurate estimation of the cannabinoid in the tested samples. The performed experiments indicate that the low response of the MS detector towards CBG results from its transformation to the pyranic CBG form, which occurs as an effect of electrons energy absorption by CBG molecules in the MS ionization chamber. The most frequent method of increasing the chromatographic analysis sensitivity to the examined compounds is their preliminary derivatization. The presented results shows that although the signal increase of CBG can be obtained through its derivatization by silylation and/or acylation, the greatest increase is observed in the case of its cyclization to the pyranic CBG form during the sample preparation process. The CBG cyclization procedure is very simple and workable in estimating this cannabinoid in blood/plasma samples.

The presented transformation of CBG to its pyranic form seems to be one of the best ways to increase the analytical sensitivity of GC–MS to CBG, but it has one disadvantage. The cyclization of CBG to its pyranic form requires an acidic environment, in which not only CBG undergoes structural transformation in such an environment, but so does CBD (to Δ^9^-/Δ^8^-THC) and Δ^9^-THC (to Δ^8^-THC). Therefore, if it is necessary to accurately determine the concentration of THC/CBD in the test sample, these cannabinoids should be determined before and then CBG using the described method. Although the cyclization process causes an additional CBD and THC transformations, nevertheless, the method has great potential in increasing the CBG signal making the quantification of this bio-marker in real samples easier.

### Supplementary Information

Below is the link to the electronic supplementary material.

Fig. 1S TICCs of DCM solutions of CBG samples exposed to thermal treatment (a), UV radiation (b) and electron stream radiation (c) (PDF 2778 KB)

Fig. 2S TICCs of Pyr-CBG and Pyr-CBG-TMS corresponding before and after derivatization (PDF 1062 KB)

Fig. 3S Possible CBG cyclization pathways in Diels-Alder reaction (PDF 292 KB)

Table 1S. 1H and 13C NMR data of CBG-4H in CDCl3 (DOCX 30 KB)

Table 2S. 1H and 13C NMR data of Pyr-CBG in CDCl3 (DOCX 29 KB)

## Data Availability

All data generated or analyzed during this study are included in this article.

## References

[1] Ramzy V, Priefer R (2021). THC detection in the breath. Talanta.

[2] Morales P, Hurst DP, Reggio PH (2017). Molecular targets of the phytocannabinoids: a complex picture. Prog Chem Org Nat Prod.

[3] Delgado-Povedano MM, Sánchez-Carnerero Callado C, Priego-Capote F, Ferreiro-Vera C (2020). Untargeted characterization of extracts from *Cannabis sativa* L. cultivars by gas and liquid chromatography coupled to mass spectrometry in high resolution mode. Talanta.

[4] Kimura T, Takaya M, Usami N, Watanabe K, Yamamoto I (2019). ∆9-Tetrahydrocannabinol, a major marijuana component, enhances the anesthetic effect of pentobarbital through the CB1 receptor. Forensic Toxicol.

[5] Dybowski MP, Dawidowicz AL (2018). Application of the QuEChERS procedure for analysis of Δ^9^-tetrahydrocannabinol and its metabolites in authentic whole blood samples by GC–MS/MS. Forensic Toxicol.

[6] Burnier C, Esseiva P, Roussel C (2019). Quantification of THC in Cannabis plants by fast-HPLC-DAD: a promising method for routine analyses. Talanta.

[7] Pichini S, Malaca S, Gottardi M, Pérez-Acevedo AP, Papaseit E, Perez-Maña C, Farré M, Pacifici R, Tagliabracci A, Mannocchi G, Busardò FP (2021). UHPLC-MS/MS analysis of cannabidiol metabolites in serum and urine samples. Application to an individual treated with medical cannabis. Talanta.

[8] Nelson KM, Bisson J, Singh G, Graham JG, Chen S-N, Friesen JB, Dahlin JL, Niemitz M, Walters MA, Pauli GF (2020). The essential medicinal chemistry of cannabidiol (CBD). J Med Chem.

[9] Devinsky O, Cilio MR, Cross H, Fernandez-Ruiz J, French J, Hill C, Katz R, Di Marzo V, Jutras-Aswad D, Notcutt WG, Martinez-Orgado J, Robson PJ, Rohrback BG, Thiele E, Whalley B, Friedman D (2014). Cannabidiol: pharmacology and potential therapeutic role in epilepsy and other neuropsychiatric disorders. Epilepsia.

[10] Crippa JA, Guimarães FS, Campos AC, Zuardi AW (2018). Translational investigation of the therapeutic potential of cannabidiol (CBD): toward a new age. Front Immunol.

[11] Tzadok M, Uliel-Siboni S, Linder I, Kramer U, Epstein O, Menascu S, Nissenkorn A, Yosef OB, Hyman E, Granot D, Dor M, Lerman-Sagie T, Ben-Zeev B (2016). CBD-enriched medical cannabis for intractable pediatric epilepsy: the current Israeli experience. Seizure.

[12] Pertwee RG, Mechoulam R (2005). Cannabidiol as a potential medicine. Cannabinoids as therapeutics.

[13] Deville M, Charlier C (2023). Cannabidiol in urine is not a proof of CBD consumption—lesson learned from urine sample analysis in routine caseworks. Forensic Toxicol.

[14] Deiana S, Preedy VR (2017). Potential medical uses of cannabigerol: a brief overview. Handbook of cannabis and related pathologies.

[15] Rague JM, Ma M, Dooley G, Wang GS, Friedman K, Henthorn TK, Brooks-Russell A, Kosnett MJ (2023). The minor cannabinoid cannabigerol (CBG) is a highly specific blood biomarker of recent cannabis smoking. Clin Toxicol.

[16] Jastrząb A, Jarocka-Karpowicz I, Skrzydlewska E (2022). The origin and biomedical relevance of cannabigerol. Int J Mol Sci.

[17] Hidvégi E, Somogyi GP (2010). Detection of cannabigerol and its presumptive metabolite in human urine after Cannabis consumption. Pharmazie.

[18] Krämer M, Schäper M, Dücker K, Philipsen A, Losacker M, Dreimüller N, Engelmann J, Madea B, Hess C (2021). Detectability of cannabinoids in the serum samples of cannabis users: indicators of recent cannabis use? A follow-up study. Drug Test Anal.

[19] Déléaval M, Burri H, Bakelants E (2022). Harmless herbs? A case report of acquired long QT syndrome and torsades de pointes in a patient taking herbal supplements. HeartRythm Case Rep.

[20] Vlad RA, Farczádi L, Toma CM, Imre S, Antonoaea P, Rédai EM, Muntean DL, Ciurba A (2021). Cannabidiol content evaluation in commercial dietary supplements and stability in oil vehicle. Stud Univ Babes-Bolyai Chem.

[21] Andrenyak DM, Moody DE, Slawson MH, O’Leary DS, Haney M (2017). Determination of ∆-9-tetrahydrocannabinol (THC), 11-hydroxy-THC, 11-nor-9-carboxy-THC and cannabidiol in human plasma using gas chromatography-tandem mass spectrometry. J Anal Toxicol.

[22] Zekič J, Križman M (2020). Development of gas-chromatographic method for simultaneous determination of cannabinoids and terpenes in hemp. Molecules.

[23] Dybowski MP, Dawidowicz AL, Typek R, Rombel M (2020). Conversion of cannabidiol (CBD) to Δ9-tetrahydrocannabinol (Δ9-THC) during protein precipitations prior to plasma samples analysis by chromatography—troubles with reliable CBD quantitation when acidic precipitation agents are applied. Talanta.

[24] Mareck U, Fusshöller G, Geyer H, Huestis MA, Scheiff AB, Thevis M (2021). Preliminary data on the potential for unintentional antidoping rule violations by permitted cannabidiol (CBD) use. Drug Test Anal.

[25] Antunes M, Barroso M, Gallardo E (2023). Analysis of cannabinoids in biological specimens: an update. Int J Environ Res Public Health.

[26] Dawidowicz AL, Dybowski MP, Rombel M, Typek R (2021). Improving the sensitivity of estimating CBD and other xenobiotics in plasma samples: oleamide-induced transient matrix effect. J Pharm Biomed Anal.

[27] Typek R, Holowinski P, Dawidowicz AL, Dybowski MP, Rombel M (2023). Chromatographic analysis of CBD and THC after their acylation with blockade of compound transformation. Talanta.

[28] Fodor B, Molnár-Perl I (2017). The role of derivatization techniques in the analysis of plant cannabinoids by gas chromatography mass spectrometry. TrAC Trends Anal Chem.

[29] Pacifici R, Pichini S, Pellegrini M, Rotolo MC, Giorgetti R, Tagliabracci A, Busardò FP, Huestis MA (2020). THC and CBD concentrations in blood, oral fluid and urine following a single and repeated administration of “light cannabis”. Clin Chem Lab Med.

[30] Newmeyer MN, Swortwood MJ, Barnes AJ, Abulseoud OA, Scheidweiler KB, Huestis MA (2016). Free and glucuronide whole blood cannabinoids’ pharmacokinetics after controlled smoked, vaporized, and oral cannabis administration in frequent and occasional cannabis users: identification of recent cannabis intake. Clin Chem.

[31] Roy P, Dennis DG, Eschbach MD, Anand SD, Xu F, Maturano J, Hellman J, Sarlah D, Das A (2022). Metabolites of cannabigerol generated by human cytochrome P450s are bioactive. Biochemistry.

[32] García-Valverde MT, Sánchez-Carnerero Callado C, Díaz-Liñán MC, Sánchez de Medina V, Hidalgo-García J, Nadal X, Hanuš L, Ferreiro-Vera C (2022). Effect of temperature in the degradation of cannabinoids: From a brief residence in the gas chromatography inlet port to a longer period in thermal treatments. Front Chem.

