# Supplementary figures and images for: Cannabigerol (CBG) signal enhancement in its analysis by gas chromatography coupled with tandem mass spectrometry
Source: Forensic Toxicol. 2023 Sep 27;42(1):31–44. doi: 10.1007/s11419-023-00673-x (PMC10808273; doi:10.1007/s11419-023-00673-x)

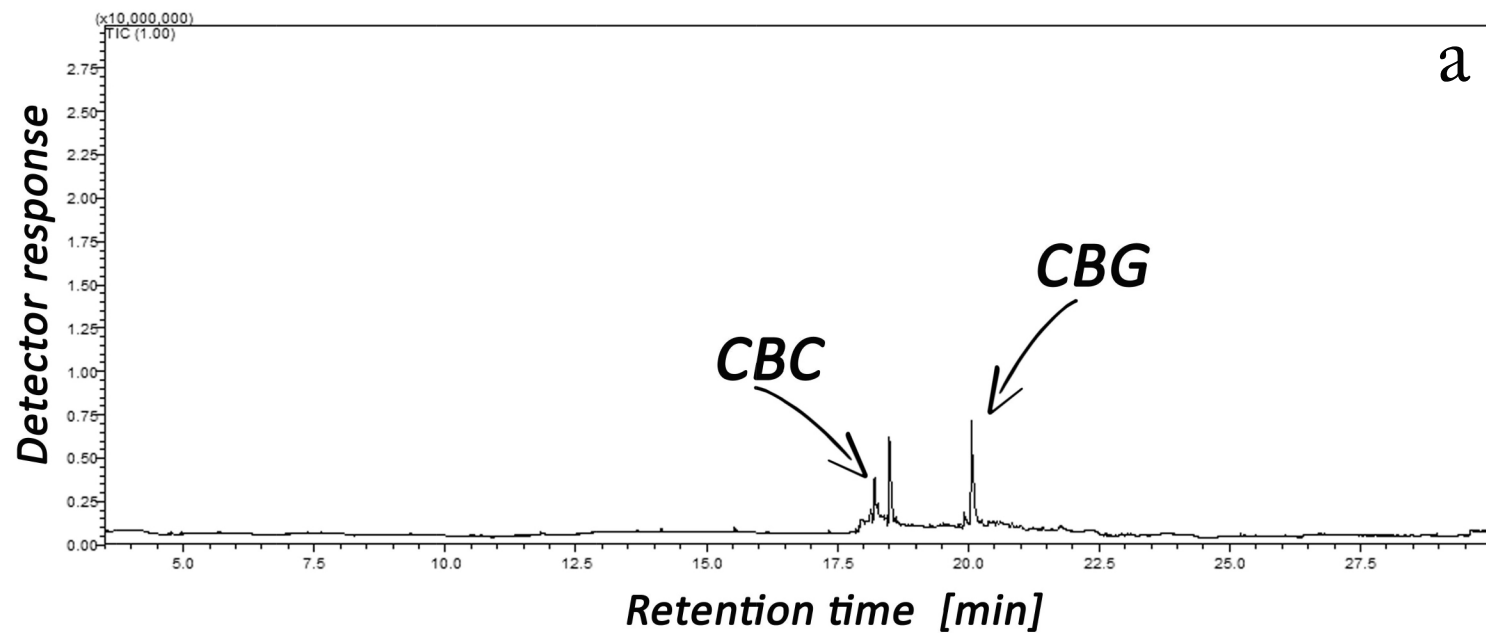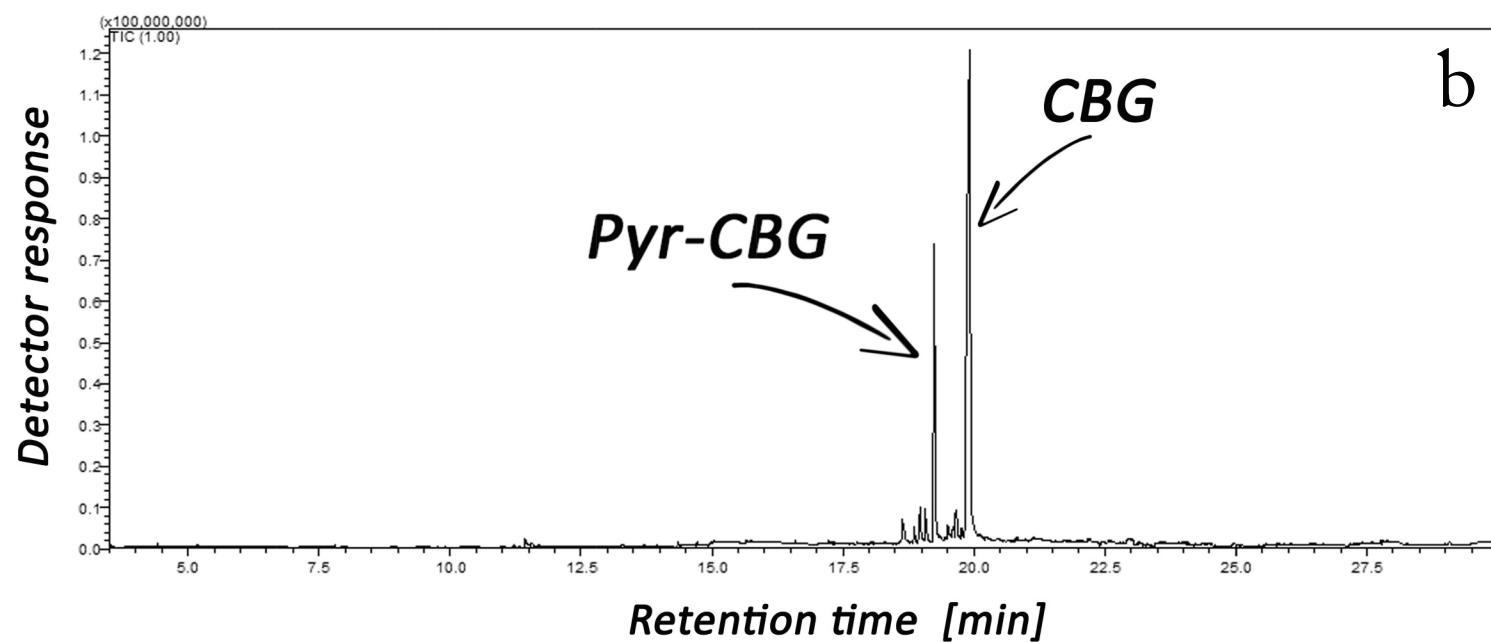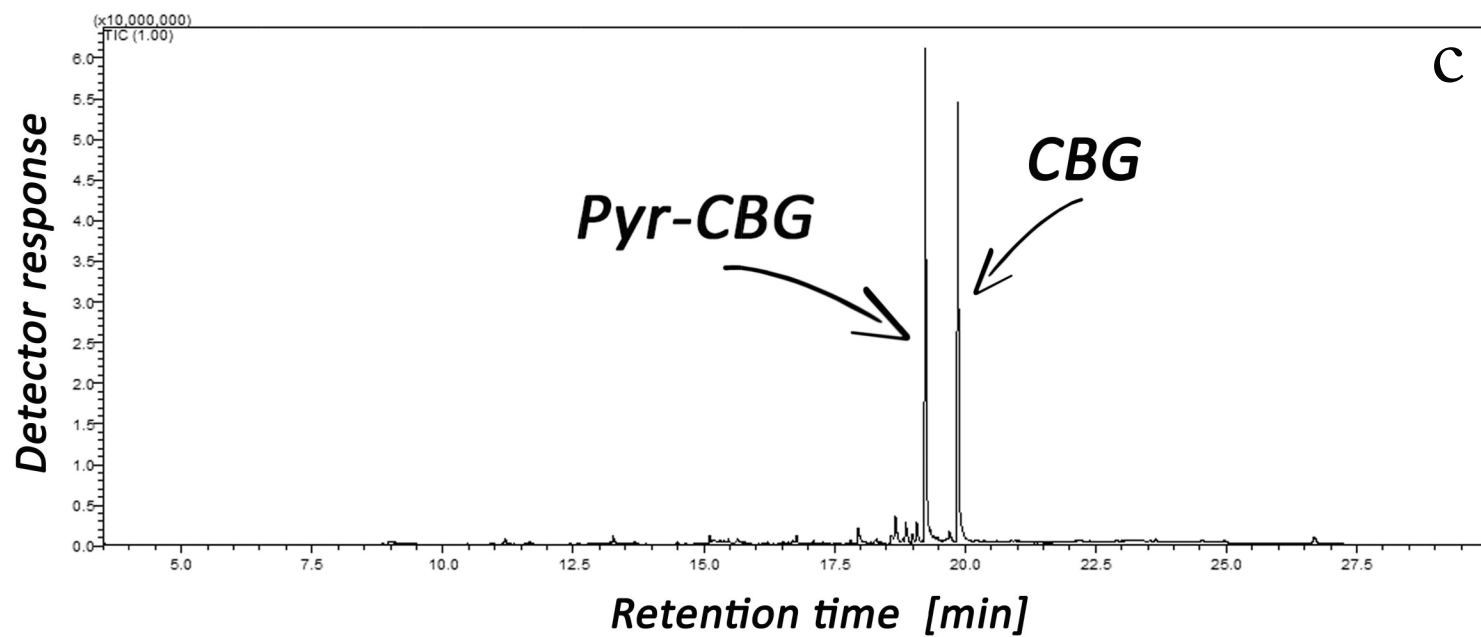

Supplement: Supplementary file 1 — Fig. 1S TICCs of DCM solutions of CBG samples exposed to thermal treatment (a), UV radiation (b) and electron stream radiation (c) (PDF 2778 KB) [file 11419_2023_673_MOESM1_ESM.pdf]

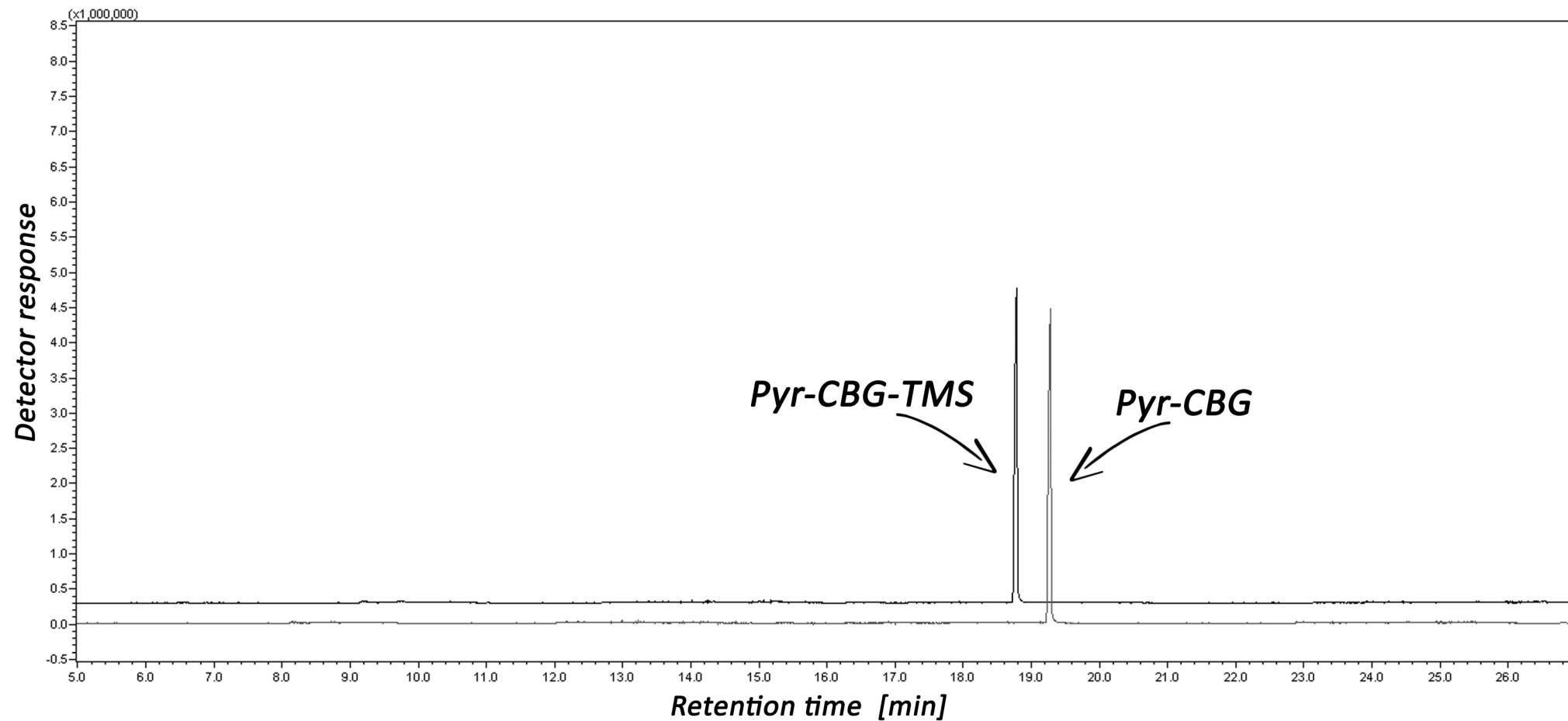

Supplement: Supplementary file 2 — Fig. 2S TICCs of Pyr-CBG and Pyr-CBG-TMS corresponding before and after derivatization (PDF 1062 KB) [file 11419_2023_673_MOESM2_ESM.pdf]

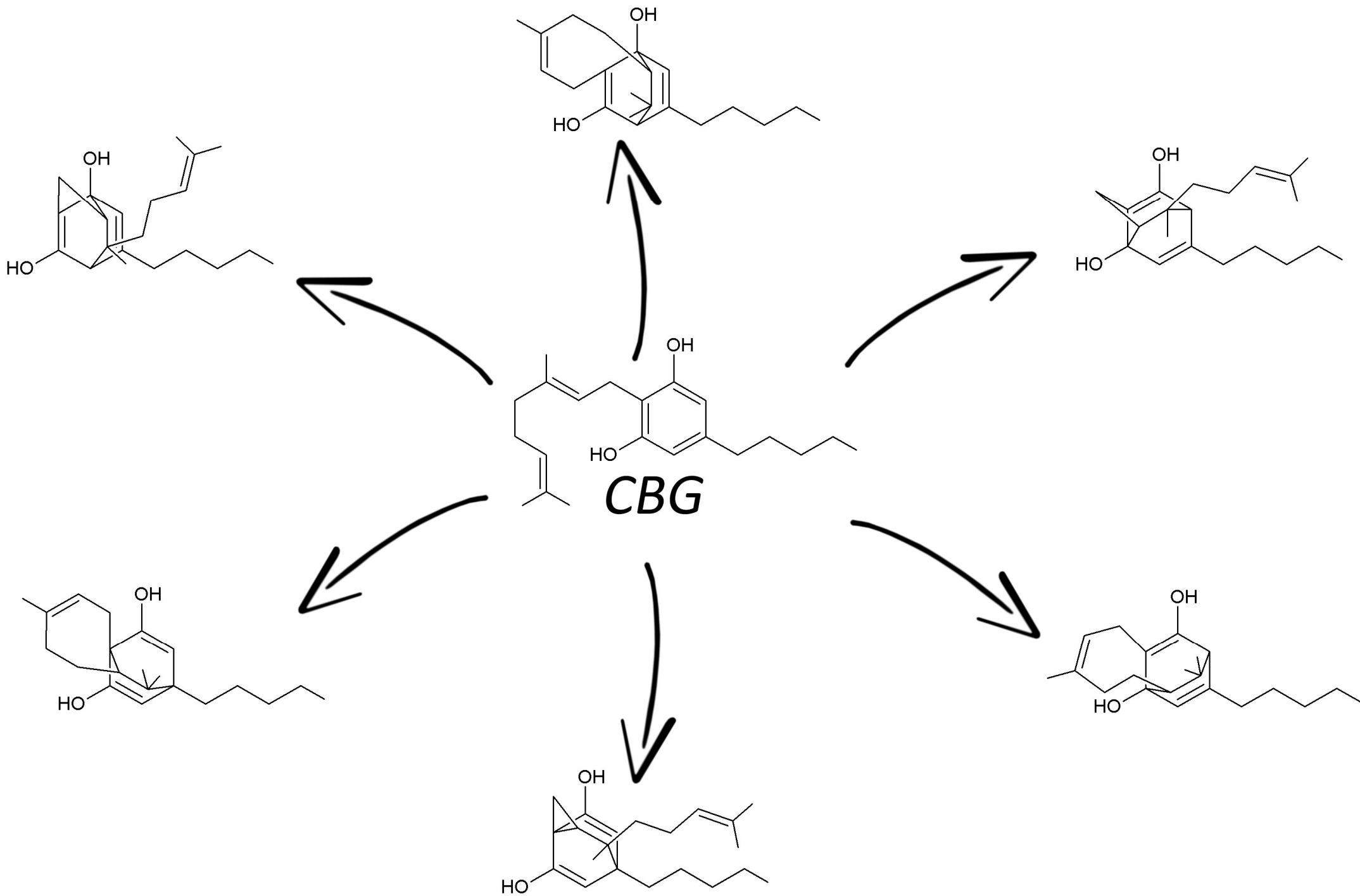

Supplement: Supplementary file 3 — Fig. 3S Possible CBG cyclization pathways in Diels-Alder reaction (PDF 292 KB) [file 11419_2023_673_MOESM3_ESM.pdf]
