# Supplementary material for: Cannabigerol (CBG) signal enhancement in its analysis by gas chromatography coupled with tandem mass spectrometry
Source: Forensic Toxicol. 2023 Sep 27;42(1):31–44. doi: 10.1007/s11419-023-00673-x (PMC10808273; doi:10.1007/s11419-023-00673-x)
Supplement: Supplementary file 4 — Table 1S. 1H and 13C NMR data of CBG-4H in CDCl3 (DOCX 30 KB) [file 11419_2023_673_MOESM4_ESM.docx]

**Table 1S.** 1H and 13C NMR data of CBG-4H derivative in CDCl_3_

|  |  | |
| --- | --- | --- |
| No. | δ_C_, type | δ_H_ (*J* in Hz) |
| 1 | 20.7, CH_2_ | 2.60, m |
|  |  | 2.54, m |
| 2 | 36.4, CH_2_ | 1.53, m^a^ |
|  |  | 1.35, m^b^ |
| 3 | 33.2, CH | 1.48, m^a^ |
| 4 | 19.6, CH_3_ | 0.95, d (6.5) |
| 5 | 37.2, CH_2_ | 1.33, m^b^ |
|  |  | 1.13, m^c^ |
| 6 | 39.4, CH_2_ | 1.12, m^c^ |
| 7 | 22.5, CH_2_ | 1.31, m^b^ |
| 8 | 28.0, CH | 1.51, m^a^ |
| 9 | 22.7, CH_3_ | 0.86, d (6.8) |
| 10 |  |  |
| 1′ | 113.0, C | - |
| 2′ | 154.4, C | - |
| 3′ | 108.0, CH | 6.22, s |
| 4′ | 142.0, C | - |
| 5′ | 108.0, CH | 6.22, s |
| 6′ | 154.4, C | - |
| 2′-OH | - | 4.97, brs |
| 6′-OH | - | 4.97, brs |
| 1′′ | 35.5, CH_2_ | 2.43, t (7.8) |
| 2′′ | 30.9, CH_2_ | 1.55, m^a^ |
| 3′′ | 31.6, CH_2_ | 1.29, m^b^ |
| 4′′ | 22.7, CH_2_ | 1.30, m^b^ |
| 5′′ | 14.1, CH_3_ | 0.88, t (7.0) |

^a-c^ Overlapping signals.
