# Supplementary material for: Cannabigerol (CBG) signal enhancement in its analysis by gas chromatography coupled with tandem mass spectrometry
Source: Forensic Toxicol. 2023 Sep 27;42(1):31–44. doi: 10.1007/s11419-023-00673-x (PMC10808273; doi:10.1007/s11419-023-00673-x)
Supplement: Supplementary file 5 — Table 2S. 1H and 13C NMR data of Pyr-CBG in CDCl3 (DOCX 29 KB) [file 11419_2023_673_MOESM5_ESM.docx]

**Table 2S.** 1H and 13C NMR data of Pyr-CBG derivative in CDCl_3_.

|  |  | |
| --- | --- | --- |
| No. | δ_C_, type | δ_H_ (*J* in Hz) |
| 1 | 16.5, CH_2_ | 2.60, td (6.8, 2.5) |
| 2 | 30.4, CH_2_ | 1.84, m |
|  |  | 1.77, m |
| 3 | 75.7, C | - |
| 4 | 24.0, CH_3_ | 1.28, s |
| 5 | 39.2, CH_2_ | 1.62, m |
| 6 | 22.3, CH_2_ | 2.09, q (7.9) |
| 7 | 124.3, CH | 5.10, m |
| 8 | 131.6, C | - |
| 9 | 25.7, CH_3_ | 1.67, s |
| 10 | 17.7, CH_3_ | 1.59, s^a^ |
| 1′ | 105.6, C | - |
| 2′ | 153.4, C | - |
| 3′ | 106.3, CH | 6.17, d (1.3) |
| 4′ | 142.5, C | - |
| 5′ | 109.8, CH | 6.27, d (1.2) |
| 6′ | 154.6, C | - |
| 2′-OH | - | 4.72, brs |
| 1′′ | 35.6, CH_2_ | 2.44, t (7.5) |
| 2′′ | 30.9, CH_2_ | 1.56, m^a^ |
| 3′′ | 31.7, CH_2_ | 1.29, m^b^ |
| 4′′ | 22.7, CH_2_ | 1.31, m^b^ |
| 5′′ | 14.2, CH_3_ | 0.88, t (7.0) |

^a-b^ Overlapping signals.
